# Euthanasia under Homœopathy

**Published:** 1896-04

**Authors:** Wm. Keaney

**Affiliations:** De Soto, Mo.


### EUTHANASIA UNDER HOMŒOPATHY.

WM. KEANEY, M. D., DE SOTO, MO.

I had, not long since, a case of tubercular laryngitis—a woman, married, æt. forty-eight years, the mother of four children, all living, who, under the influence of Iodine, and later on, of Bromine, I had kept very comfortable, though getting weaker, of course, every day.

As she approached the "jumping-off place" she was tortured by inability to swallow; even water would cause coughing, strangling, blueness, and great exhaustion; mouth and throat became very sore and painful. AM-CAUST.[20] helped her at once, and life was bearable again; she could eat and drink with comparative comfort. Her brother, an allopathic physician, came to visit her, and, although she was feeling moderately well, he had to *do* something, and, as he found her heart was weak, he pumped Digitalis into her as a heart tonic. The dysphagia and dyspnœa returned, and after three days of torture, unable to swallow even a teaspoonful of water, she died.

Apropos of euthanasia, have you ever exhibited Cuprum? I had four years ago a young man who had grafted a severe attack of syphilis into a tubercular diathesis, and living a very rapid life, soon came to the end of his journey. Sulph. helped his diarrhœa, Kali-c. his cough and chest pains, at the various times they were indicated; so he kept going all the time with a good appetite, good sleep, and comfortable days, save when disturbed by the diarrhœa, or pains in chest and cough.

The last two days he lived had lost his voice, coughed hollow, rattling; couldn't get up anything. Enormous ecchymoses appeared all over extremities. Cup-met. stopped the cough, which was incessant, eased the dyspnœa, kept him conscious, let him talk (in a whisper), and up to the last minute he was conscious, dying with the words, "I'm so sleepy I want to rest."

You'll notice I gave my last case Sulph., notwithstanding the fact that a certain faction of homœopathic doctors allege that though indicated, Sulph. is *dangerous* (!) to give, "it will kill the patient," "cause suppuration," and other kindred calamities. That is arrant nonsense, and will keep no one, I hope, from giving at all times, places, and circumstances, the indicated remedy, whether it be Sulph., Phos., Sil., or any other remedy called for by *all* the symptoms. To let a certain set of real or fancied pathological phenomena deter us from giving the simillimum, is to annul the law of cure. "False in one, so in all." Both experience and reason contravene this addition to the *homœopathic* law. There is no doubt but that Phos., Sulph., and others have done injury in consumption and similar degenerative disease conditions, but I am sure that they were not well indicated, only seemingly so; that it is quite possible in these diseases for an imperfect selection to do harm, is, I presume within the range of your experience.

My dear Doctor, possibly I am "making faces" at one of your pet beliefs (though I do not think so), but if I am, give us an editorial on the subject, and I'll come back at you to your sorrow.

